# Mr. Syme on the Treatment of Ulcers

**Published:** 1830-04-01

**Authors:** 


### EDINBURGH SURGICAL HOSPITAL.*

#### TREATMENT OF ULCERS.

Mr. Syme continues to follow up his very laudable plan of reporting the more interesting cases that occur to him at his surgical hospital. None can appreciate more highly than we do, the advantages of surgeons to public institutions thus laying open to their brethren the stores of professional information which their practice affords them; and none have awarded the meed of honest approbation more earnestly or more constantly than we have done. But we cannot conceal from ourselves, and will not conceal from our readers, that reports from what we may term *private* hospitals offer a temptation to puffery and even quackery; and, more than that, the records of medicine prove but too surely and sadly, that what is coarsely called humbug, of the worst description, has been practised within their walls and promulgated without them. We make these remarks to put Mr. Syme upon his guard, to warn him from falling into an error to which the weak parts of human nature might possibly incline him, to render him in fact respectable and respected, useful to the profession, and in the long run to himself. We take this opportunity of stating that nothing of so degrading a stamp has characterised or characterises Mr. Syme's reports, but we cannot avoid remarking a leaning on his part towards his own institution, which though perfectly excusable, must yet be deemed a weakness. We remarked in our review of his last, that when a little older as a hospital surgeon, he would become a little more distrustful of hospital cases and hospital cures. We may now observe that he should not lean so fondly as he seems to do on the gratitude and so forth of the class of persons with whom he will have to do. Unless human nature is vastly different in the modern Athens and the modern Babylon, the thanks of hospital patients are too often but a sorry farce. It appears that Mr. Syme is a very successful chirurgeon in the treatment of sore legs, and what is equally gratifying, his pupils would seem to have caught no small portion of that "divine ichor," which, like the blood from the wounded Deity in the Iliad, distils from their worthy preceptor's clinical instructions.

"The very great number and variety of ulcers which have been presented at the hospital, afforded me ample opportunity of illustrating the treatment of these most frequent and distressing affections. I felt much satisfaction in doing so on several accounts. 1*st*. The great relief derived by the patients, many of whom were restored to the means of earning a livelihood, of which they had previously been long deprived. 2*d*. The useful instruction derived by my pupils, who were wise enough to regard the ordinary duties of their profession as deserving of at least as much attention as those great and bloody exploits, which occur but seldom in the course of practice. 3*d*. The widely extended and sound reputation acquired by the new Institution from curing diseases, where the relief is so great and manifest, and the means of remedy are so little disagreeable."

The *sound* reputation of the institution for *bad* legs is no doubt a very flattering thing. Let us see the secret, which, like all very valuable things, is contained in a mighty small compass:

"It would be out of place for me to enter here into a detail of cases which are usually reputed trifling and uninteresting, though there are few surgical diseases which more seriously affect the patient's comfort; but I cannot refrain from reporting at some length, and with some care, the advantages of a practice which I have lately introduced in their treatment and which I believe is a new one.

In treating what are called Indolent ulcers of the leg, I used to regard the plan recommended by Mr. Baynton as approaching nearly to perfection, and still believe that, when properly executed, it will sooner or later effect a cure, if a cure be practicable; but

* Ed. Med. and Surg. Journ. No. 102.

another method has lately suggested itself to me, which seems in many respects preferable.

It is not unusual to meet with cases of indolent ulcers which, after exhibiting their characteristic obstinacy in opposition to the most careful treatment, heal up at once without any attention, so soon as the limb begins to recover from an attack of phlegmonous erysipelas which it has happened to suffer. The observation of such cases led me to try the effect of inducing a similar inflammation artificially, and the result has fully equalled my expectations. The means employed for this purpose were blisters, and the object being to excite a smart and diffuse inflammation, they were not limited in extent to the size of the sore, but were made to cover a great part of the limb, and were allowed to remain in operation for a long while, sometimes even twenty-four hours.

The first effect of the blisters in these cases *is a more than ordinary inflammation and discharge,* the surface sometimes continuing to suppurate profusely for several days, just as if the cutis had been denuded by a scald or burn.

In a day or two the patient is agreeably surprised by observing that the œdematous swelling of the limb, which so constantly accompanies ulcers of the kind under consideration, begins to subside, and in the course of a very short time, rarely exceeding a week or two, it nearly or entirely disappears. The consequence of this detumescence is a great diminution in the size of the sore, which also comes to be on a level with the surrounding skin. Then the surface takes on a healthy granulating appearance, and the sore heals, partly by contraction, partly by the formation of a cicatrix. For the first few days after the blister has been applied, some simple ointment may be used, just as in the ordinary treatment of a blistered surface, and afterwards a wash of acetate of lead or sulphate of zinc, in the proportion of one or two grains to the ounce. If the sore should again prove obstinate, the blister may be repeated, and if a small part remains stationary towards the conclusion of the cure, it ought to be filled with the red oxide of mercury, or a mixture of this powder with flour. My friend, Professor Davidson of Aberdeen, induced me to try this application in the treatment of ulcers, and I cannot say too much in its praise, especially in the case just mentioned. After one or two dressings it forms a firm crust over the sore, which ought not to be disturbed, and renders any farther interference unnecessary.

I have no hesitation in ascribing the good effect of blisters which have been just described to their stimulating the absorbent vessels, so as to remove the œdema. We know that blisters possess a singular power of doing this, as is exemplified in the cure of dropsies of the joints and bursæ, and it is easy to see that the existence of œdema must be an insuperable obstacle to the healing of the ulcer. It prevents the contractile effect of the granulating action, and thus occasions a struggle, which probably gives rise to the pain and other symptoms that so often induce a resemblance between indolent and irritable ulcers. And we find, in fact, that all the modes of treating the ulcers in question which have ever proved serviceable, such as the horizontal posture, the roller of Underwood and Whateley, or the adhesive straps of Baynton, tend to reduce œdematous swelling. Some frivolous and wrong-headed improvers have advised that the straps should not encircle the whole limb, but only two-thirds of it, and in so far have done what they could to bring themselves and the practice into contempt. I lately cured an ulcer on the leg of a lady which had existed without interruption for twenty years, and was deemed incurable, because it had resisted the most assiduous exertions of several surgeons in town. When I proposed to apply adhesive plasters, the patient would hardly consent, because they had been tried previously without success. I ascertained that they had not encircled the limb, and hence the failure."

As Mr. Syme has been so obliging as to communicate this "secret worth knowing" to *us,* we will hint another equally valuable to *him:* viz. that he will find old ulcers on the legs just as troublesome in 1839 as he did in 1829, before he employed the blistering practice, or leased the surgical hospital. Whenever there are a thousand specifics for a disease, the man of the world may be certain there are none. There is only one remedy for the itch, because any one may cure it with brimstone; there are at least a hundred for tetanus, because no man can cure it all! Mr. Stafford published an octavo volume the other day to assure the public that melted wax was almost infallible for the cure of ulcers. The sanguine were in extasies; "Ευρηκα" was the general cry; but we doubted. Surely we were right, for here comes Mr. Syme "in a month, a little month," with as dismal a tone as ever on the obstinacy of ulcers, save under *his* new method. Each inventor of a system or a remedy is like Janus; one face, a very long one, looks back on all former systems and remedies, the other, bedecked with wreathed smiles, regards the inventor's sunny Utopia. For ourselves, cold, cautious and calculating, we look before we leap, wait before we pronounce, hesitate before we believe. Probably before our next number sees the light, another certain cure for ulcers will have been discovered to be necessary, and necessarily discovered.

